# Complementary AI in higher education: behavioral, cognitive, and ethical implications of ChatGPT and DeepSeek

**DOI:** 10.3389/fpsyg.2026.1699114

**Published:** 2026-03-24

**Authors:** Eman Faisal

**Affiliations:** Department of Curriculum and Instruction, College of Education, King Saud University, Riyadh, Saudi Arabia

**Keywords:** artificial intelligence, ChatGPT, DeepSeek, higher education, mixed methods

## Abstract

The present mixed-methods study integrates a systematic review of 51 studies (2024–2025) with qualitative findings from eight participants, who were asked to complete a semi-structured questionnaire, to comprehend the complementary functions, pedagogical tasks, and ethical concerns associated with ChatGPT and DeepSeek in tertiary education. ChatGPT enhances creativity, language learning, and motivational participation, while DeepSeek excels in analytical accuracy and domain-specific performance, particularly in STEM and medical education. Evidence from both phases indicates that their integration underpins a twin-function pedagogy that couples generative fluency and systematic reasoning. Yet threats still exist, such as hallucination-induced errors, academic fraud, and a decline in critical thinking in the absence of metacognitive scaffolding and ethical regulation. Six thematic categories—personalized education, academic honesty, metacognition, model performance, adoption patterns, and domain-specific use—emerged in parallel with three cross-cutting patterns highlighting cognitive trade-offs, equity-related matters, and neurobehavioral dynamics. Together, these results suggest that interdisciplinary cooperation is crucial in developing AI–human co-regulation models, ethics-informed curricula, and deployment scenarios tailored to diverse environments. ChatGPT and DeepSeek are not rival technologies but complementary cognitive agents whose prudent integration can enhance creativity, accuracy, and ethical resilience in higher education.

## 1 Introduction

The integration of Generative Artificial Intelligence (GenAI) technologies, specifically ChatGPT and DeepSeek, has not only brought about a technological shift but also a profound change in cognitive processes, behavioral regulation, and moral decision-making in higher education (Yusuf et al., 2024). Large language models (LLMs) assume the role of cognitive co-creators, revolutionizing how students can access self-regulated learning, exercise critical thinking, and engage with ethical concerns such as academic honesty and plagiarism (Rudolph et al., 2024). Empirical studies highlight their complementary nature: as ChatGPT enhances creativity and engagement in language learning and writing, DeepSeek is unparalleled in technical disciplines like programming and medicine due to its analytical precision and cost-effectiveness (Jin et al., 2025; Rudolph et al., 2024). This complementarity announces a paradigm where proprietary and open-source models complement different learning needs.

Even when promising, the fragmented literature prevents a comprehensive interpretation of how these tools differentially affect psychological constructs (e.g., creativity, metacognition, ethical resilience) and behavioral outcomes (e.g., dishonesty, overreliance, equity in access) across disciplines (e.g., ChatGPT for English as a Second Language - ESL writing). Mohamed (2023); and DeepSeek in social science [e.g., Dilshad et al. (2025)], excluding systematic comparisons of their domain-specific effectiveness, behavioral risks, and equity concerns. For instance, ChatGPT’s effectiveness in personalized tutoring is balanced by risks of deterioration in critical thinking and academic dishonesty (Dandage, 2025). In contrast, DeepSeek’s reliability in technical work addresses context-specific inaccuracies and cultural biases (ibid.). Furthermore, there are gaps within evidence-based systems regarding the inhibition of hallucinations (In AI language models, hallucination refers to outputs that appear coherent but are factually incorrect, unsupported, or fabricated, such as non-existent article references or inaccurate definitions), plagiarism, and cognitive exclusion—problems that need to be addressed urgently through behavioral science lenses such as Self-Regulated Learning (SRL) and ethical decision-making.

Along similar lines, the rapid digital transformation in the Saudi Arabian higher education context has accelerated experimentation with AI without unified institutional policies governing its ethical use, assessment boundaries, or faculty training (Alotaibi and Alshehri, 2023; Faisal, 2024). Meanwhile, national initiatives promote innovation; AI governance frameworks remain uneven across universities, creating another chasm between adoption and pedagogical regulation. It is this contextual tension that motivates the present study beyond literature-driven gaps.

### 1.1 Theoretical and conceptual background

#### 1.1.1 Generative AI in higher education

Generative Artificial Intelligence (GenAI) has rapidly transformed higher education, creating new opportunities for cognitive support beyond traditional teaching (Alotaibi and Alshehri, 2023; Yusuf et al., 2024). Unlike other educational technologies, which are often viewed as cognitive automation tools, large language models have been shown to be cognitive agents with dialogic explanatory capacities, thereby providing feedback and scaffolding. Studies have conceptualized Generative Artificial Intelligence as a form of cognitive augmentation, distinguishing it from cognitive automation.

#### 1.1.2 Cognitive offloading, metacognition, and SRL

From a cognitive perspective, the use of GenAI raises crucial concerns about cognitive offloading, self-regulated learning (SRL), and the nature of metacognitive awareness (Tomisu et al., 2025). While aids to cognition are beneficial for improving process efficiency and reducing unnecessary load, limiting reliance on such aids is thought to hinder reflective control and strategic processes. In AI-mediated learning environments, learners’ dependence is often linked to how they organize, follow up on, and reflect on their learning processes.

#### 1.1.3 Ethical decision-making and academic integrity

The ethical issues with GenAI in higher education include academic integrity, authorship confusion, and epistemic responsibility. This differs from conventional plagiarism tools, as GenAI raises questions about authorship and guidance, thereby complicating governance and ethics for higher education bodies (Gulumbe et al., 2025). Ethical decision-making in education discusses the need for disclosure norms and moral agency regarding GenAI.

#### 1.1.4 Complementarity and dual-process pedagogy

Recent discussions of conception suggest that AI models may exhibit distinct cognitive affordances, rather than uniform abilities and faculties (Luo and Yusuf, 2025). Drawing on the theoretical basis of dual-process learning, fluency and precision may be understood as separate yet mutually complementary pedagogic roles, providing a basis for exploring and analyzing ChatGPT and DeepSeek as distinct learning facilitators rather than as equally aligned, competitive tools. The difference between ChatGPT and DeepSeek, as found in this research, is not based on an evaluation of their architectures, as access to their complete training datasets and optimization processes is not publicly available. The basis for comparing these two tools is the patterns of pedagogical performance documented empirically.

### 1.2 Positioning the present study

Drawing on these conceptual foundations, the present study distinguishes between theoretical framing and empirical synthesis: the former concerns the theoretical background, and the latter concerns the empirical synthesis stage. However, the systematic review conducted in Stage One of the present study provides evidence-based thematic generation.

This systematic review addresses these shortcomings by synthesizing 51 studies to address three core questions. Prioritizing the domains of psychology, neurocognition, and organizational behavior, this review emphasizes rigorous empirical synthesis, replicable findings, and actionable strategies for leveraging the educational potential of AI.

The current study tries to answer the following research questions:

RQ1: In what ways do ChatGPT and DeepSeek differentially impact pedagogical and cognitive-behavioral functions across disciplines?

RQ2: What are the ensuing behavioral and ethical implications of their use, and how do they vary by sociocultural context?

RQ3: What evidence-based strategies enhance synergies between ChatGPT and DeepSeek to optimize metacognitive vigilance and ethical resilience?

This review offers educators, policymakers, and institutions actionable frameworks to leverage the complementary strengths of ChatGPT and DeepSeek, striking a balance between personalized innovation and ethical safeguards. The findings advance behavioral science by mapping cognitive-ethical trade-offs in AI adoption while offering reproducible strategies for domain-specific implementation.

## 2 Materials and methods

This study employed a sequential, mixed-methods approach with a qualitative-dominant design. In the first stage, a systematic literature review examined the pedagogical, ethical, and cognitive-behavioral dimensions of generative AI tools, specifically ChatGPT and DeepSeek, within the context of university learning environments. The emergence of themes during this stage provided direct input into the development of the semi-structured questionnaire used in the second stage, which collected qualitative data from participants with first-hand experience of the AI models. The design ensured that the questionnaire probed problems by drawing upon empirical literature, while also permitting the validation, explication, or denial of theoretical patterns identified in the review. The sequential approach, as represented in qualitative dominance, resonates well with the traditions of exploratory mixed-methods approaches in which Stage One of the sequential mixed-methods design serves as inductive thematic synthesis and Stage Two serves as contextual elaboration and theoretical refinement through experiential insight (Creswell and Plano Clark, 2007; Tashakkori and Teddlie, 2008). It is worth noting that Stage Two was not designed for statistical validation or generalization.

### 2.1 Stage one (systematic review)

#### 2.1.1 The aim and design

This phase aims to synthesize empirical studies on the pedagogical functions, cognitive-behavioral implications, and ethical concerns associated with the use of ChatGPT and DeepSeek in university educational contexts. The PRISMA 2020 guidelines (Page et al., 2021) were followed in conducting this systematic review to ensure transparency, reproducibility, and methodological quality throughout the identification, screening, eligibility, and inclusion phases. The review considered peer-reviewed journal articles and conference papers indexed in three prominent academic databases: Web of Science (WOS), EBSCO, and ProQuest. These databases were selected due to their high indexing quality and broad interdisciplinary coverage, particularly in education, psychology, and the behavioral sciences, as well as their suitability for capturing studies that investigate domain-specific teaching applications, integration challenges, and synergy approaches for implementing generative AI tools. All searches were applied to titles, abstracts, and full texts, with the final database search conducted on June 29, 2025.

To qualify, the research had to be empirical, published in English, and conducted between January 2024 and June 2025. Each study had to expressly examine ChatGPT and/or DeepSeek within the higher education context and discuss at least one of three basic analytical dimensions: the pedagogical purposes these tools support (e.g., personalized tutoring, writing support, or skills development), the challenges to their implementation (e.g., ethical risks, hallucinations, or unequal access), or strategies that enable their effective and complementary integration across disciplines. This ensured that the review focused on contemporary, research-driven insights that reflect the evolving educational and behavioral-cognitive impact of GenAI.

Studies were excluded if they focused on K–12 educational settings, examined AI models other than ChatGPT or DeepSeek (such as Gemini, Claude, or Copilot), or presented highly technical findings (e.g., engineering optimization models) without direct pedagogical application. Also excluded were studies that did not report behavioral outcomes (e.g., academic honesty, reliance, cognitive load management) or cognitive-ethical dimensions (e.g., critical thinking, metacognitive development, ethical reasoning). In this manner, there was a keen focus on research that facilitated meaningful engagement with the intersection of pedagogy, behavioral-cognitive processes, and ethics in the use of generative AI in higher education.

#### 2.1.2 Search strategy and study selection

**Figure 1:**
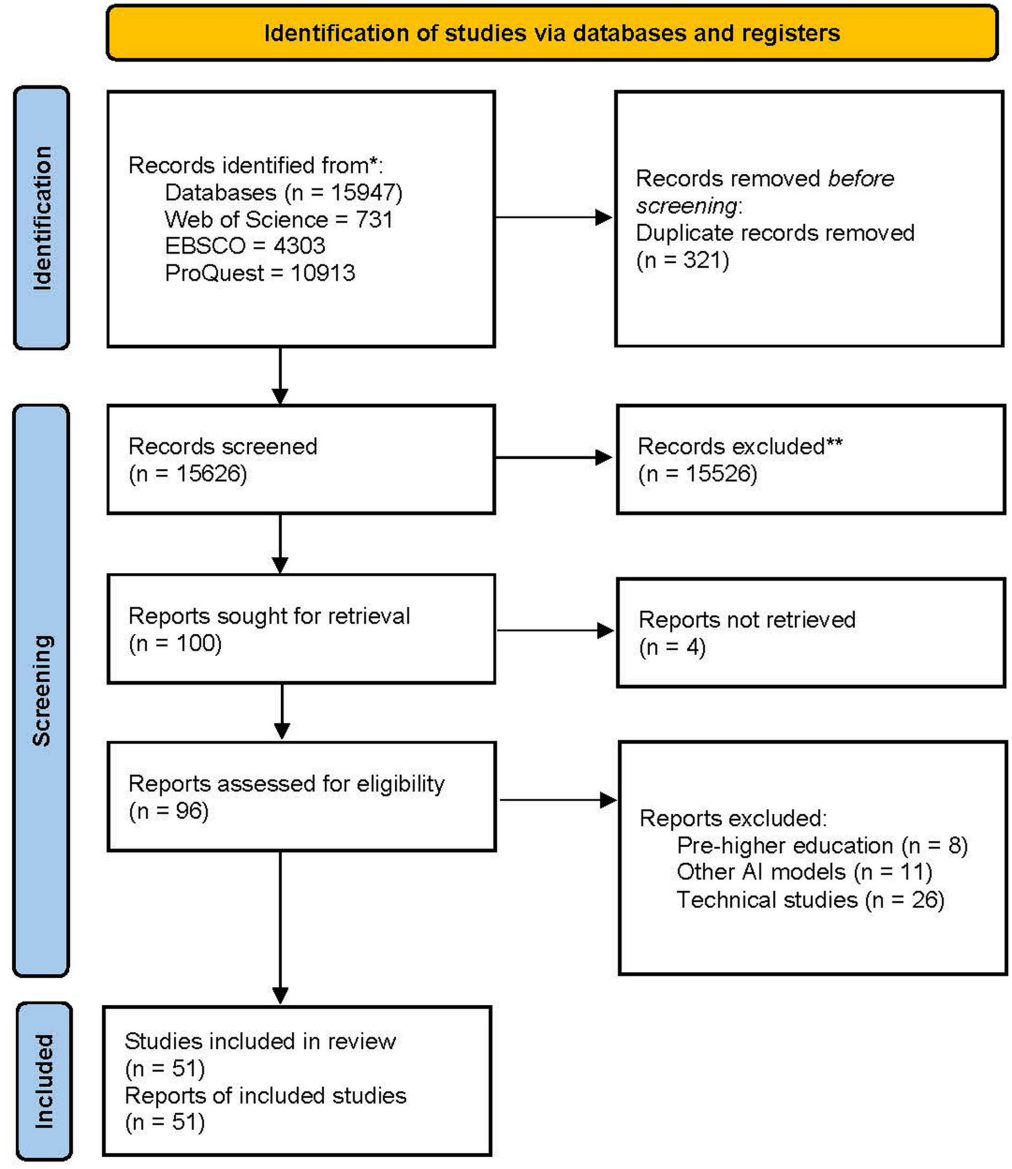
PRISMA flowchart.

Initial searches for “DeepSeek” yielded minimal results (WOS: *n* = 185; EBSCO: *n* = 221; ProQuest: *n* = 295), reflecting its emergent status in academic literature. Subsequent searches for “ChatGPT” produced substantially more publications (WOS: *n* = 546; EBSCO: *n* = 4,082; ProQuest: *n* = 10,618). The second search used the combination of “ChatGPT” AND “academic integrity” OR “critical thinking” AND “higher education,” showing that (WOS: *n* = 248; EBSCO: *n* = 1,462; ProQuest: *n* = 6,689). However, when using the same combination of these terms, except that ChatGPT was replaced with DeepSeek, the studies were notably scarce (WOS: *n* = 44; EBSCO: *n* = 83; ProQuest: *n* = 165). The last searches did not target each AI model solely. However, it was modified to be “ChatGPT and DeepSeek” AND “academic integrity” OR “critical thinking” AND “higher education,” showing that (WOS: *n* = 11; EBSCO: *n* = 7; ProQuest: *n* = 33). In total, the final literature corpus comprised 51 studies that addressed the behavioral, cognitive, and ethical components. Figure 1 presents the PRISMA 2020 Flow Diagram, highlighting the identification, screening, eligibility, and inclusion of studies for the literature synthesis. Specifically, the final literature corpus comprised 51 studies, reflecting the inclusion criteria that prioritized depth over quantity.

#### 2.1.3 Data analysis

Qualitative content analysis was applied to explore the extracted data (Mayring, 2002) using ATLAS.ti version 25.0.1. Primary codes were developed to encompass the comparative capabilities of ChatGPT and DeepSeek, as well as their pedagogical roles, cognitive and behavioral impacts, and implementation problems. Regularities, divergences, and trends were created through repeated comparative analysis of coded parts. Secondary thematic clusters coded findings into six conceptual categories (e.g., personalized learning, academic integrity, development, and metacognitive growth).

The derived themes were used to purposively construct the semi-structured questionnaire for Stage Two, with the qualitative stage inquiring into the identical behavioral, cognitive, and ethical constructs identified in the literature. This sequential design allowed the research to shift from the derivation of theoretically motivated themes to participant validation and extension. Results (Section 3) present findings grouped by thematic categories and analytical dimensions, founded on evidence-based behavioral and pedagogical implementation plans. The six final thematic categories were retained based on frequency, conceptual saturation, and theoretical relevance across both pedagogical and behavioral-cognitive dimensions.

### 2.2 Stage two (semi-structured questionnaire)

#### 2.2.1 The aim and design

This phase aimed to build directly on the findings of Stage One by gathering first-hand experiential data to research how users experience and interact with ChatGPT and DeepSeek within educational contexts. It was designed to validate and expand the themes identified in the systematic review, thereby linking theoretical insights with lived experiences. This qualitative step helped to gain insights into participants’ explanations about using both AI models.

#### 2.2.2 Sampling

A purposive sampling strategy was employed to obtain participants’ perspectives on behavioral, cognitive, and ethical aspects of using ChatGPT and/or DeepSeek in learning and education. The sample consisted of eight AI experts (five females and three males), all of whom had over 10 years of experience (one had less than 5 years of experience). The participants were recruited from different institutional environments, i.e., public universities, private institutions, and an education consultancy. The selection process was based on the author’s academic relationships with lecturers and professors who work in colleges and departments of computer and information sciences, as well as on their availability.

The sample size of eight participants was deemed appropriate for an in-depth qualitative study, allowing for thematic saturation and rich exploration of cognitive-behavioral experiences (Ando et al., 2014; Coenen et al., 2012; DiCicco-Bloom and Crabtree, 2006; Guest et al., 2006). Saturation was indicated when no new codes or conceptual insights emerged across successive responses. Saturation in the present study was conceptual rather than statistical, with no new thematic dimensions emerging after the sixth response. The emphasis was on analytic depth and theoretical resonance rather than generalization to the population. Ethical approval for data collection was obtained from the university’s Research Ethics Committee (ethical approval reference number: KSU-HE-25-915). All participants were 18 years of age or older. Written consent was obtained from each participant, and each participant was referred to in the results section as P1 or P5. Table 1 shows the participants’ demographic information. The author acknowledges her positionality as an academic within the Saudi higher education system, which informed both access to participants and interpretive sensitivity during analysis.

**Table 1:** Demographic information of the participants.

Participant	Gender	Qualification	Experience	Sector of word
P1	M	PhD	More than 10 years	Academic
P2	F	PhD	More than 10 years	Academic
P3	M	PhD	More than 10 years	Academic
P4	M	PhD	More than 10 years	Academic
P5	F	Master	More than 10 years	Government
P6	F	Bachelor’s	More than 10 years	Academic
P7	F	Bachelor’s	More than 10 years	Government
P8	F	Master	Less than 5 years	Private

#### 2.2.3 Semi-structured questionnaire and procedures

A semi-structured, open-ended questionnaire was developed, comprising two parts. The first part was to gather demographic information (i.e., gender, qualification, experience, and the sector in which you currently work). The second part was explicitly informed by the thematic categories identified in Stage One, ensuring that the questions reflected the behavioral, cognitive, and ethical dimensions of AI in higher education. This part included four open-ended questions designed to explore participants’ behavioral, cognitive, and ethical experiences with ChatGPT and DeepSeek:

- How do you use the program (e.g., translation, search, writing, etc.)?
- In your opinion, how can the combined use of ChatGPT and DeepSeek improve university learning?
- In your opinion, what are the most critical risks or problems that might arise from using AI tools in university education, like ChatGPT and DeepSeek?
- In your opinion, how can we help students think more deeply and avoid relying entirely on artificial intelligence?

The electronic version of the questionnaire (Google Forms) was sent via WhatsApp, which is the most used messaging application in Saudi Arabia (Alharbi et al., 2025). The questionnaire was pilot tested with two scholars to check the clarity of wording, and the average completion time was 10 min. Feedback from pilot participants identified minor ambiguities in wording and question flow, leading to small refinements that enhanced clarity and coherence without altering the instrument’s conceptual structure. The data were sorted into segments with similar content to facilitate analysis.

#### 2.2.4 Data analysis

Participants’ responses were analyzed through thematic analysis to confirm, develop, or disprove the themes generated in Stage One. Braun and Clarke’s (2021) six-stage thematic analysis approach was adopted: (1) familiarization with the data, (2) code generation, (3) searching for themes, (4) inspection of themes, (5) definition and naming of themes, and (6) writing up the report. ATLAS.ti 25.0.1 was used to build the master codes, which were cross-checked against the themes developed in the systematic review to validate, refine, or expand them. Two research assistants independently coded a subset of responses to enhance reliability, and an interpretive meeting was held between the research assistants and the researcher to reach a consensus on the structure of the findings. Disagreements were resolved through iterative interpretive discussion and consensus-building, which served as the primary strategy for establishing analytic rigor, consistent with qualitative epistemological traditions.

### 2.3 Data integration

Data integration was performed after the independent analysis of both phases, utilizing a side-by-side comparison strategy. Findings from Stage One served as the baseline themes, which were then juxtaposed with participants’ insights to identify points of convergence, divergence, and extension. This process enabled triangulation of evidence, enriched the interpretation of results, and ensured that practical voices validated or nuanced the theoretical claims. Integration was explicitly aligned with each research question, highlighting behavioral, cognitive, and ethical dimensions, thereby strengthening the coherence and applicability of the conclusion. This integration strategy is one of methodological complementarity rather than numerical triangulation. The qualitative phase was used to refine the theoretical synthesis but was not intended as a confirmatory or statistically generalizable phase (Table 2).

**Table 2:** Summary of stage one thematic synthesis.

Key findings	Methods	Representative studies	Theme
ChatGPT supports personalization; DeepSeek improves analytic accuracy	Systematic review; quasi-experimental	(Dilshad et al., 2025; Lo et al., 2024)	(1) Personalized Learning and Cognitive Support
Ethical risks require institutional regulation	Surveys; policy analysis	(Chemaya and Martin, 2024; Johnston et al., 2024)	(2) Academic Integrity and Ethics
AI is effective only with SRL scaffolding	Pre-post-tests; correlational	(Mahapatra, 2024; Shi et al., 2025)	(3) Metacognitive Development
Task-specific performance superiority	Benchmarking	(Kerimbayev et al., 2024; Zuzanna et al., 2025)	(4) Model Performance and Pedagogy
Adoption mediated by culture and trust	UTAUT modelling	(Cabero-Almenara et al., 2025)	(5) Adoption and Sociocultural Context
Human judgment remains essential	Applied case studies	(Bhattacharya et al., 2025)	(6) Domain-Specific Implementation

## 3 Results

### 3.1 Stage one (systematic review)

This systematic review synthesizes 51 studies (2024–2025) to answer three research questions. ChatGPT excels at creative ideation and motivational engagement, while DeepSeek excels at analytical accuracy and cognitive efficiency. These instructional advantages are countered by significant risks, including hallucination-based inaccuracies, undermining critical competencies, and equity-related adoption barriers. For optimal outcomes, it entails a combination of ChatGPT’s generative flexibility and DeepSeek’s structured rigor, supported by metacognitive scaffolding and ethical guardrails to mitigate over-reliance and foster critical thinking.

These findings are organized into the following six structured themes. An overview of these themes, along with representative studies, methods, and key findings, is summarized in Table 3.

**Table 3:** Integration of stage one and stage two findings

Theme	Stage one: systematic review	Stage two: qualitative findings	Integration/interpretation
(1) Personalized learning and cognitive support	ChatGPT excels in creative ideation and motivation; DeepSeek excels in analytical accuracy; AI supports individualized instruction, but requires oversight (Dilshad et al., 2025; EL Fathi et al., 2025; Faisal, 2024; Huang et al., 2025; Lo et al., 2024; Runceanu et al., 2025; Umar et al., 2025; Wang, 2025; Yang et al., 2025)	Participants highlighted AI’s flexibility in explaining complex concepts, providing multiple perspectives, and supporting diverse student needs (P2, P3, P6)	Strong convergence: Both stages confirm that AI tools enhance personalization and reduce cognitive load, though careful instructional design is needed.
(2) Academic integrity and ethics	Risks of over-reliance, plagiarism, and hallucinated content; need for institutional policies and ethics training (Cheng et al., 2025; Johnston et al., 2024; Mahapatra, 2024; Nelson et al., 2025; Ozfidan et al., 2024; Patil et al., 2025; Rudolph et al., 2025; Zhang and An, 2025)	Participants expressed concerns about copyright, misinformation, and erosion of critical thinking (P1, P4, P7)	Strong convergence: Ethical risks are consistently observed; qualitative data contextualize them with practical classroom concerns.
(3) Metacognitive development	AI scaffolds learning but cannot replace reflective and self-regulated learning; iterative feedback enhances higher-order thinking (Alafnan, 2025; Kotsis, 2025; Lin et al., 2025; Mahapatra, 2024; Maltseva and Pavlova, 2025; Mohammad et al., 2025; Naznin et al., 2025; Shi et al., 2025; Smiju and Adinath, 2025)	Participants emphasized using AI as a tool to prompt reflection, comparison, and critical analysis (P2, P5, P8)	Convergence: Both stages highlight AI as a facilitator rather than a replacement for metacognitive engagement.
(4) Model performance and pedagogy	Task-specific performance varies; DeepSeek is more precise for STEM/technical content, ChatGPT is stronger in creativity; caution against overreliance (Al-Jarf, 2025; Dandage, 2025; Dilshad et al., 2025; Kerimbayev et al., 2024; Mayank et al., 2025; Özcivelek and Özcan, 2025; Ramlogan et al., 2025; Sallam et al., 2025; Spennemann, 2025; Zuzanna et al., 2025)	Participants appreciated timesaving and idea-generation benefits but noted the need for human verification due to errors or hallucinations (P4, P6, P7)	Complementarity: Quantitative review confirms model strengths and limits; qualitative data illustrate real-world application and the need for teacher mediation.
(5) Adoption and sociocultural context	Adoption is influenced by performance expectancy, infrastructure, trust, and cultural norms (Abdulrahman et al., 2025; Cabero-Almenara et al., 2025; Jorge et al., 2025; Lin et al., 2025; Liu et al., 2025; Martínez et al., 2025; Wu, 2025)	Participants reported uneven institutional policies, technical issues, and varied faculty attitudes affecting adoption (P1, P3, P8)	Convergence with contextual expansion: Both stages highlight social and structural barriers, with the participants’ answers providing nuanced, lived perspectives.
(6) Domain-specific implementation	AI is more effective in general knowledge; high-stakes or specialized domains require human oversight (Bhattacharya et al., 2025; Casey et al., 2025; Kotsis, 2025; Lo et al., 2024; Martínez et al., 2025; Ramlogan et al., 2025; Stollberger et al., 2025; Wen-Hsiu et al., 2025; Yilmaz et al., 2025; Zhu and Yu, 2025)	Participants reported variable reliability depending on discipline, with technical areas requiring careful verification (P2, P5, P6)	Strong convergence: Domain specificity matters; AI is a complementary tool requiring human judgment in specialized areas.

#### 3.1.1 Theme 1. Personalized learning and cognitive support

This theme explores how AI personalizes learning to cater to individual cognitive needs. Lo et al. (2024) conducted a systematic review to critically examine 70 empirical studies on ChatGPT in ESL/EFL instruction, identifying its potential to support personalized learning activities and teacher assistance, while also implicating significant risks of academic dishonesty and over-reliance on writing. Similarly, Faisal (2024) reviewed Saudi higher education and found ChatGPT to be a game-changer in remodeling teaching approaches, activating students, bridging language divides, and fostering inclusivity, aligning with national ambitions for academic excellence. Umar et al. (2025) demonstrated how RAG-augmented LLMs enhance medical students’ journal club experiences by providing tailored summaries and answers to questions, thereby improving preparation and the quality of discussion.

In technical domains, Huang et al. (2025) proposed a rigorous evaluation framework using Delphi and AHP methods to assess AI-generated content, prioritizing “authenticity,” “accuracy,” and “relevance” as quality indicators. Yang et al. (2025) applied location-based AI vocabulary training for Japanese learners, dynamically adjusting content to their geographical context, thus enhancing sustainable learning. Dilshad et al. (2025) compared DeepSeek and ChatGPT in social science education, finding that DeepSeek’s detailed explanations led to 25.9% higher post-test scores compared to 17.7% for ChatGPT, emphasizing the latter’s better capability for individualized academic assistance.

Challenges persist regarding the balance between automation and cognitive development. EL Fathi et al. (2025) demonstrated that ChatGPT enhanced thermodynamics conceptual knowledge through constructivist questioning but left quantitative misconceptions unresolved, highlighting limitations in fostering deep thought. Wang (2025) advocated for the use of generative AI (specifically DeepSeek) in programming courses, proposing adaptive teaching models while cautioning against motivational erosion. Runceanu et al. (2025) introduced an open-source AI framework enabling offline personalized learning, validated across STEM disciplines. Synthesizing these findings, AI’s pedagogical synergy lies in scaling individualized instruction while requiring vigilant oversight to prevent dependency.

#### 3.1.2 Theme 2. Academic integrity and ethics

This subject theoretically discusses behavioral norms and policy responses to AI ethics. Johnston et al. (2024) surveyed 2,555 university students and identified that 70.4% opposed using ChatGPT for entire essays and 41.1% demanded institution-based policies, indicating an acute awareness of ethical boundaries. Rudolph et al. (2025) also denounced Silicon Valley’s “hype,” arguing widespread AI adoption erodes academic honesty and evidence-based instruction, and advocating for critical AI literacy courses. Chemaya and Martin (2024) uncovered errors in detection: under low usage for grammar checking, ChatGPT was routinely marked as dishonest by AI detectors, which complicated enforcement.

Cross-cultural findings reveal contradictory attitudes. Nelson et al. (2025) surveyed the views of Ecuadorian students and found that they considered unreported AI-generated writing academically dishonest, but not when human proofreading was employed. Zhang and An (2025) reported that Chinese graduate students envisioned ChatGPT as a tool requiring “human agency” for oversight. Ozfidan et al. (2024) noted that Saudi students experience trust issues despite the use of AI tools in academic writing, highlighting cultural gaps in trust. Mahapatra (2024) documented the risks associated with relying on ESL writing interventions, recommending ChatGPT as a means for formative feedback, rather than a crutch.

Solutions emerge in collaborative frameworks. Cheng et al. (2025) proposed guidelines addressing hallucinations and fabricated references, emphasizing the need for transparent disclosure. Patil et al. (2025) advocated ethics training for medical educators to navigate AI-assisted assessments responsibly. Collectively, abstracts emphasize that ethical governance must strike a balance between innovation and behavioral safeguards, positioning institutions as key arbiters of responsible use.

#### 3.1.3 Theme 3. Metacognitive development

Research in this area investigates the impact of AI on higher-order competencies. Mahapatra (2024) employed pre- and post-tests with ESL students, demonstrating that the formative feedback provided by ChatGPT significantly improved writing ability but potentially hindered SRL. Maltseva and Pavlova (2025) compared DeepSeek with comparable tools for the acquisition of analytical skills, concluding that iterative AI feedback enhanced students’ ability to improve arguments but required teacher mediation. Shi et al. (2025) found that strong SRL was correlated with higher writing performance and wellbeing in AI-mediated environments, proving metacognition’s mediating role.

Domain-specific skills transfer varies. Alafnan (2025) benchmarked ChatGPT’s style creativity against DeepSeek’s structure precision in commercial communication, illustrating that both tools enhance human skill capabilities. Kotsis (2025) tested ChatGPT and DeepSeek on physics misconceptions and found that only explanatory dialogue corrected misunderstandings when prompts engaged reflection. Naznin et al. (2025) systematically examined the application of ChatGPT in coding teaching, achieving efficiency gains but issuing warnings about the potential for atrophy of debugging skills in the absence of regulation. Smiju and Adinath (2025) recognized ChatGPT’s conversational reasoning over DeepSeek’s technocratic problem-solving and suggested hybrid solutions.

Lin et al. (2025) revealed that 70% of Chinese learners utilized GenAI but lacked the necessary skills, citing training deficits in the internalization of capability. Mohammad et al. (2025) demonstrated that generative AI enhances diagnostic skills among medical students through virtual patient interactions, while emphasizing the need for human verification. Both results agree on a simple observation: AI scaffolds foundational competencies effectively but cannot foster metacognitive profundity outside pedagogical incorporation.

#### 3.1.4 Theme 4. Model performance pedagogy

This is a comparison of functional performance on learning tasks. Kerimbayev et al. (2024) compared 10 artificial intelligence systems, with GPT-4 ranking highest in producing creative materials, and DeepSeek ranking highest in financial-value technical support for learning computer science. Zuzanna et al. (2025) compared GPT-4o and DeepSeek-R1 on Poland’s infectious diseases exam, where DeepSeek achieved 73.85% compared to GPT-4o’s 71.43%, although both exceeded the 60.5% threshold. Dilshad et al. (2025) reported that DeepSeek scored 25.9% higher on social science tests compared to ChatGPT, due to its improved depth of explanation.

Technical and linguistic abilities differ markedly. Al-Jarf (2025) experimented with AI translation of Arabic grammatical metaphors, reporting that DeepSeek was superior to Google Translate but still struggled to translate polysemous words. Sallam et al. (2025) scored ophthalmology questions translated into Arabic and English and found that DeepSeek achieved accuracy comparable to that of GPT-4 but exhibited a slight English bias. Ramlogan et al. (2025) experimented with dental exams, and ChatGPT-4/4o achieved 77–78% accuracy but lacked clinical reasoning, despite outperforming students. Özcivelek and Özcan (2025) found that field-specific dental instruments surpassed the models in prosthesis questions.

Performance limits persist. Mayank et al. (2025) showed that Claude/DeepSeek/Grok achieved 93% accuracy with blood physiology MCQs but cautioned against overreliance. Dandage (2025) architecturally contrasted ChatGPT’s generative universality with that of DeepSeek’s computational frugality. Spennemann (2025) contrasted citation accuracy, showing ChatGPT’s tendency to “confabulate” referenceable citations. These findings highlight that task-specific utility—not universal excellence—should dictate model selection.

Synthesizing these studies, an emergent pattern suggests that there is no universal superiority in model performance across educational contexts, but rather significant contingency. ChatGPT exhibits relative strengths in generative, language-intensive, and ideational model performance, whereas DeepSeek invariably excels in analytically constrained, technical, and domain-specific model performance. Moreover, with respect to these models, there is no assumption of universal superiority across educational, pedagogical, and instructional contexts, suggesting that instructional effectiveness is not determined by the model used but rather by the contingencies.

#### 3.1.5 Theme 5. Adoption and social-cultural context

This theme examines the behavioral factors that influence the adoption of AI. Cabero-Almenara et al. (2025) applied UTAUT2 modeling in Costa Rica, with “performance expectancy” identified as the leading driver of ChatGPT adoption. Liu et al. (2025) associated with “uncertainty avoidance” and social influence barriers, namely for teacher trainees. Jorge et al. (2025) surveyed Ecuadorian students, associating the ethical use of AI with reflective thinking rather than frequency of use.

Cross-cultural differences influence implementation. Li et al. (2025) found that Hong Kong students exhibited ambivalence: 54% viewed AI as a threat to jobs, but 68% accepted it for educational purposes. Faisal (2024) identified ChatGPT as a driver of workforce readiness in Saudi Arabia, contrasting with the results of Ozfidan et al. (2024), who found that Saudi students expressed mistrust in the reliability of AI. Martínez et al. (2025) evaluated GenAI for Spanish cybersecurity translation, which highlighted accuracy-accessibility trade-offs that generated skepticism.

Training and equity concerns are dominant. Lin et al. (2025) demonstrated that 50% of Chinese learners were GenAI deficient, despite extensive use, suggesting that institutions need to upskill their staff. Wu (2025) advocated for democratizing DeepSeek for coding education but referenced infrastructure gaps. Abdulrahman et al. (2025) revealed DeepSeek’s privacy advantages in healthcare but cautioned against potential regulatory loopholes. Abstracts overall indicate that adoption is mediated through socio-culturally responsive anxiety, training, and equity approaches.

#### 3.1.6 Theme 6. Domain-specific implementation

This subject evaluates domain-specific implementation problems. Lo et al. (2024) highlighted ChatGPT’s strength in ESL composition but its weakness in speech and listening competence, suggesting a need for a balanced language mix. Bhattacharya et al. (2025) applied DeepSeek to surgical 3D reconstructions and ChatGPT to clinical decision-making, highlighting the indispensable role of human judgment. Yilmaz et al. (2025) tested 8 LLMs on questions of oral pathology and found that ChatGPT-o1 was exceptionally good (96% accuracy) but weak at case-based reasoning.

In the applied sciences, Zhu and Yu (2025) employed DeepSeek-V3 to simulate stakeholders’ perceptions of tourism education, aiming to enhance industry-oriented pedagogy. Casey et al. (2025) applied transfer learning to distribute AI training with the assistance of energy coefficients, thereby positioning nodes most optimally. Wen-Hsiu et al. (2025) compared the financial data analysis capabilities of ChatGPT-4 to those of Stata and documented equivalence in inferences but variance in implementation.

Enduring gaps continue in high-stakes domains. Ramlogan et al. (2025) once again drew attention to the limitations of LLMs in keeping pace with clinical judgment, despite test mastery. Martínez et al. (2025) observed “weird and funny” errors in AI translation of cybersecurity alerts. Kotsis (2025) illustrated that physics misconceptions reappeared in the absence of SRL cues. Stollberger et al. (2025) recommended longitudinal studies on AI-human interaction. These studies once again reiterate that domain accomplishment hinges on complementary human-AI roles, wherein AI provides scalability, and humans maintain contextual rigor.

### 3.2 Cross-cutting patterns (stage one)

Throughout the distinct thematic domains explored, this review identifies three enduring, cross-cutting patterns that reveal how ChatGPT and DeepSeek, in combination, shape educational outcomes. These patterns illustrate a shared design in how these AI models impact cognition, behavior, and equity across specific disciplines or contexts.

First, the tension between generative fluency and analytic precision is a recurring theme throughout research. ChatGPT, through its creativity and engagement, tends to evoke learner motivation and ideation, but at times masks conceptual gaps beneath surface fluency. DeepSeek, on the other hand, offers robust analytical depth, especially in STEM and clinical fields, but can produce more limited outputs requiring human mediation to decipher or repurpose. These traits, working in tandem, form a dual-cognitive system: ChatGPT evokes divergent thinking, and DeepSeek supports convergent analysis. This trend underscores the importance of task-specific alignment and hybrid deployment in optimizing learning gains while minimizing cognitive trade-offs.

Secondly, there is a consensus in many studies on the importance of metacognition and human regulation. Across all domains, AI tools enhance performance only when situated within pedagogies that are structured to enhance reflection, monitoring, and ethical decision-making. Lacking instructional scaffolds—such as guided prompts, teacher-mediated feedback, or self-regulated learning (SRL) portfolios—students used AI passively, risking overreliance and surface processing. This suggests that the productive use of AI has more to do with the quality of the cognitive and instructional environment within which it is situated, rather than the model itself.

Third, the review highlights a pervasive tension between access and equity, particularly in terms of institutional preparedness and cultural trust. While open-source frameworks like DeepSeek offer under-resourced environments scalable and affordable solutions, challenges persist—from technical literacy inequalities to socio-cultural skepticism. The commercial advantages of ChatGPT are generally outweighed by its cost, content bias, or limited cross-linguistic relevance. Variegated by regions, ethical interests, infrastructural incongruities, and unequal training opportunities shape adoption patterns, making it clear that pedagogical innovation with AI must be accompanied by policies that actively reduce epistemic and infrastructural inequalities.

Taken together, these trends suggest the need for a paradigm in which AI is not merely adopted but co-orchestrated—technologically innovative yet pedagogically aware, ethically responsible, and sensitive to learners’ cognitive and sociocultural contexts.

### 3.3 Stage two (qualitative findings from the semi-structured questionnaire)

The qualitative portion consisted of eight participants (three males and five females) who completed a semi-structured questionnaire. They all had experience with AI technologies, such as ChatGPT and DeepSeek, before participating. The participants represented a variety of academic and professional settings, including four doctoral-level, two master’s-level, and two bachelor’s-level. Seven had more than 10 years of experience, and one had less than 5 years. Five worked in higher education, two in government, and one in a private-sector technology and consulting firm.

Thematic analysis revealed six themes closely aligned with those of Stage One.

#### 3.3.1 Theme 1. Personalized learning and cognitive support

Both participants uniformly credited ChatGPT and DeepSeek for successfully explaining complex concepts and offering personalized assistance for students. Several referred to the tools’ ability to divide abstract or technical terms into basic terms.

A participant noted, “ChatGPT explained a complicated topic to my students as if they were junior middle school pupils—it made the concept instantly clearer” (P3). Another observed, “The tools give me multiple explanations for one concept, so students who do not understand the first way can still grasp it through another” (P6). A third emphasized, “Sometimes they even help me brainstorm new approaches to teaching, which saves preparation time” (P2).

These quotations highlight how AI’s flexibility enables teachers to tailor learning experiences, alleviate cognitive overload, and cater to students’ diverse needs. Participants also emphasized that these tools should supplement, rather than replace, traditional pedagogy. Such participant perspectives directly triangulate with the Stage One findings regarding the benefits of a personal learning experience that AI-assisted explanation has on cognitive clarity.

#### 3.3.2 Theme 2. Academic integrity and ethics

Ethical concerns were a consistent thread throughout the participants’ answers, echoing Stage One findings on risks of over-reliance, deceptive content, and misuse. Most users end up using these tools only as sources of information, which weakens their critical thinking skills.

One participant noted, “The majority of users tend to lean on these tools alone as sources of information, which erodes critical thinking skills” (P1). Another raised concerns, stating, “I worry about copyright and intellectual property. If the model produces text I reuse, who owns it?” (P4). A third observed, “There is also the risk of biased or false information, which students may accept without verifying” (P7).

These perceptions reflect teacher concerns about plagiarism, content authenticity, and transparency, reinforcing the importance of official AI policies and ethical application. The obvious similarity to the concerns in Stage One literature indicates that ethical issues such as overreliance, authorship, and misinformation are commonly addressed.

#### 3.3.3 Theme 3. Metacognitive development

Participants emphasized that AI should enhance, not hinder, reflective learning and critical thinking. They noted that authentic pedagogical value lies in using AI as a stepping stone toward more substantial involvement, rather than an endpoint.

One participant stated, “We need more practical exercises and live discussions to make students think more and rely less on automated tools” (P8). Another explained, “If students only copy what AI generates, they miss the chance to practice reflection and analysis” (P5). A third noted, “I encourage them to compare AI answers with textbooks—the process of noticing differences makes them more aware of their thinking” (P2).

The above passages suggest that AI, when paired with deliberate instructional design, can be a force to promote metacognition — instructing students to question, revise, and build upon information, rather than merely consuming it. These findings align with Stage One findings, which suggest that AI has a positive impact only within a context of metacognitively scaffolded pedagogies that actively promote reflection, monitoring, and self-regulation of learning.

#### 3.3.4 Theme 4. Model performance and pedagogy

Respondents discussed applying the functional aspect of AI to make teaching more effective while tolerating the disparate quality of products in various disciplines.

One participant observed, “It reduces the time it takes to search through literature and helps me generate new ideas for student projects” (P6). Another noted, “When I am designing lectures, I can use the tools to draft outlines or examples that I refine later” (P4). A third explained, “Sometimes it even provides alternative questions for exams or class discussions, which sparks creativity” (P7).

These reflections highlight AI’s dual pedagogical benefits: saving time in content preparation and offering multiple avenues of teaching, while underscoring the importance of teacher mediation. Consistent with Stage One results, these accounts again emphasize that model effectiveness is task-dependent rather than universal, thereby supporting hybrid pedagogical use.

#### 3.3.5 Theme 5. Adoption and sociocultural context

Barricades to adoption were not necessarily due to the tools themselves, but rather to structural, institutional, and cultural contexts.

One participant noted, “We do not have a unified institutional policy, so each department has its own informal rules” (P3). Another observed, “Internet disruptions and technical issues sometimes discourage people from using these tools at all” (P1). A third explained, “Some colleagues embrace AI enthusiastically, while others completely reject it—there is no shared culture yet” (P8).

These accounts illustrate an uneven adoption landscape, shaped by policy inconsistencies, infrastructure limitations, and faculty attitudes, which influence how students engage with AI technologies. This is a direct reflection of the research priorities in Stage One that highlight the fact that the adoption of AI is mediated by “sociocultural trust, institutional readiness, and policy coherence rather than merely technical capability itself.”

#### 3.3.6 Theme 6. Domain-specific implementation

Participants noted that AI effectiveness varies by discipline, with technical or specialized subjects requiring more verification.

One participant stated, “AI tools are more accurate in general knowledge areas, but when I use them for advanced domain-specific topics, I need to verify every detail” (P5). Another explained, “For technical subjects, the models often miss the precision needed, so I cannot rely on them fully” (P2). A third observed, “In the humanities, however, they provide helpful perspectives and save me time in developing examples” (P6).

The extracts suggest that disciplinary context matters: AI is a complementary tool in specialized areas, with human judgment remaining essential. These findings parallel Stage One evidence indicating the necessity of domain-specific AI implementation, with human verification and expertise, particularly in high-stakes or technically constrained domains.

### 3.4 Cross-cutting (stage two)

Running across all the themes, three cross-cutting patterns became evident, consistent with Stage One:

- Dual role of AI tools: Both excellent aids to learning and possible vectors of exposure.
- Need for balanced integration: AI needs to complement, not replace, traditional pedagogical practice.
- Centrality of training and awareness: Overwhelmingly supported formal training in AI literacy, ethics, and critical thinking as minimum necessities to utilize effectively.

### 3.5 Integration of findings of stages one and two

This section synthesizes the findings from Stage One (systematic review) and Stage Two (qualitative data from a semi-structured questionnaire), highlighting areas of convergence, divergence, and complementarity. By integrating these two stages, the study provides a comprehensive understanding of the pedagogical roles, challenges, and contextual factors associated with ChatGPT and DeepSeek in higher education (Table 3).

The twin-function pedagogy construct is not the result of opinions gathered in the study but is the outcome of the alignment between the thematic patterns in the literature and the experiential opinions offered by the participants. The two stages of the study reveal a similar pattern in participants’ opinions: the ChatGPT model is associated with generative fluency and motivational engagement, while the DeepSeek model is associated with analytic precision and logical reasoning.

Despite strong convergence, meaningful divergences emerged. Although strong convergence in results was noted across both stages, there were also pronounced divergences: some studies viewed ChatGPT as a catalyst for reflective learning, whereas others reported negative effects on critical thinking from students’ heavy use of AI technology, even in the absence of instructors.

## 4 Discussion

This research integrated data from 51 studies and eight expert answers to examine ChatGPT and DeepSeek in tertiary education contexts. Through an integrated cognitive-behavioral and pedagogical approach, both models offer paradigmatic educational potential. Still, successful implementations must be calibrated to particular domains, supervised by neuroethics, and socio-culturally contextualized. Five main findings ensue: pedagogical complementarity, behavioral-ethical trade-offs, metacognitive scaffolding, contextual adoption barriers, and implications for AI-infused instructional design.

### 4.1 Pedagogical complementarity: GenAI as a double-role cognitive agent

The strongest insight is the complementary advantages between ChatGPT and DeepSeek. ChatGPT possesses excellent generative fluency and creative interaction, facilitating divergent thinking and motivational activation (Alafnan, 2025; Lo et al., 2024), whereas DeepSeek possesses analytical rigor and domain-specific precision, facilitating structured reasoning (Dilshad et al., 2025; Maltseva and Pavlova, 2025). They form an opposing-function pedagogical system: ChatGPT enables ideation, and DeepSeek offers precision. Systematic reviews and semi-structured questionnaires attest that such collaboration enhances learning outcomes, reduces misconceptions, and advances hybrid pedagogical models in which AI is calibrated to the task, subject matter, and learner profiles.

### 4.2 Ethical trade-offs and behavioral risks

While each model has advantages, each also presents challenges in terms of behavior and ethics. Among the dangers are hallucination-induced errors, loss of critical thinking, and over-reliance, particularly if students lack AI literacy or reflective guidance (Chemaya and Martin, 2024; Spennemann, 2025). Naturalness on the part of ChatGPT can mask knowledge gaps, and accuracy on the part of DeepSeek may discourage participation (Wang, 2025). Stage two findings validated these issues and emphasized the importance of approaching AI as augmentation, rather than automation, and of building curricular safeguards against cognitive passivity and ethical disengagement.

### 4.3 Metacognitive scaffolding: the mission link

Metacognition then became the key mediator to the effective use of AI. Without explicit scaffolding (such as Reflective Prompting, Explain-Back training, or SRL portfolios), AI risks bypassing deeper schema assimilation (Kotsis, 2025; Shi et al., 2025). Both stages underscored that AI is most effective when it becomes part of pedagogies actively cultivating reflection, analysis, and ethical sensitivities. Future applications should continue co-regulatory AI-human architectures, such as neuroadaptive feedback calibrated to learners’ reflective and cognitive capacities.

### 4.4 Sociocultural apprehension, equity discrepancies, and institutional obligations

Sociocultural settings, disparity gaps, and infrastructural readiness strongly influence AI adoption. Barriers include economic constraints (Li et al., 2025), a lack of trust (Ozfidan et al., 2024), and perceptions regarding the legitimacy of the tool (Zhang and An, 2025). Open-source technologies, such as DeepSeek, can help mitigate disparities but require institutional support, clear documentation, and context-based training. Both the review and the participants’ answers emphasize the institution’s intervention to ensure the inclusive adoption of AI and promote cross-cultural capacity development.

### 4.5 From AI integration to AI partnership

The argument favors moving beyond integration to co-designed AI partnerships. ChatGPT and DeepSeek should not only augment curricula but also co-design learning experiences with and for students and faculty. Successful implementation is all about neuroethical literacy, reflective practice, and institutional design, securing innovation balance with ethical oversight (Rudolph et al., 2025; Smiju and Adinath, 2025; Umar et al., 2025).

Although this paper mentions certain AI models in its discussion, its main contribution lies in developing a theory of complementary cognitive affordances in large language models. The twin-function framework has been designed to remain conceptually relevant across model versions, as it relates to a broader mechanism of generative fluency and analytic structuring in AI-mediated pedagogy.

### 4.6 Theoretical and practical implications

#### 4.6.1 Theoretical implications

There are the following theoretical implications of the integration of empirical findings on ChatGPT and DeepSeek in higher education:

- Cognitive-Affective Dualism: ChatGPT may be conceptually associated with affective-motivational engagement, whereas DeepSeek aligns with analytic cognitive processing, promoting creative interaction, whereas DeepSeek engages dorsolateral-analytic processes, enhancing organized thinking. This justifies dual-process learning theories in AI-mediated environments.
- AI-Augmented SRL and Ethical Vigilance: Self-regulated learning (SRL) and ethical metacognition serve as the mediating variable between AI usage and learning outcomes, transforming reflection into a distributed co-regulatory process.
- Paradigm Shift in Instructional Roles: Cognitive co-creators are AI models, breaking with traditional instructor-centered paradigms and empirically substantiating post-humanist educational theory.

#### 4.6.2 Practical implications

From these theoretical implications, the following practical recommendations can guide the application of AI in higher education:

- Model Matching: Utilize ChatGPT for ideation and creativity-driven disciplines, and DeepSeek for STEM or precision-sensitive disciplines.
- Instructional Integration: Embed AI in flipped learning, peer review, and SRL workflows; use prompt engineering and reflective checkpoints to enable student engagement. Examples include explain-back tasks where students justify AI-generated responses in their own words, reflective AI-use journals documenting decision points, and instructor-guided comparison activities between AI outputs and disciplinary sources.
- Equity and Access: Support open-source alternatives, provide localized training, and supply infrastructure support for resource-limited learners, aligned with global frameworks such as UNESCO’s AI in Education guidelines
- Ethics and Policy: Develop AI literacy curriculum and ethics-by-design solutions, including institutional audits and alignment rubrics. Ethics-by-design can be operationalized through mandatory AI disclosure statements, assessment rubrics that distinguish assistance from authorship, and institutional AI audits embedded in course design.

### 4.7 Strengths, limitations, and future trajectories

#### 4.7.1 Strengths

This study is one of the first mixed-method syntheses of ChatGPT and DeepSeek in higher education. Integrating cognitive neuroscience, behavioral ethics, and pedagogical practice, it provides a robust theoretical foundation. In addition, cross-domain and cross-cultural considerations promote generalizability, while neurocognitive framing enhances theoretical contributions.

#### 4.7.2 Limitations

Nevertheless, five limitations are worth noting: (1) Language and Indexing Bias: Only English-language articles indexed in Western-dominated databases (WOS, EBSCO, ProQuest) were included, which may limit cultural representativeness and exclude region-specific or non-English scholarship, particularly from the Global South. (2) Emergent Status of DeepSeek: The availability of longitudinal data is also scarce, and the volume of classroom-based research on DeepSeek remains in development. As such, the findings related to the comparison between DeepSeek should be viewed with caution, pending further empirical substantiation. Given the emergent nature of DeepSeek in the context of education research, it is important to note that the associated research base is less well-developed than that related to ChatGPT. (3) Temporal Scope: Research was limited to January 2024–June 2025, potentially excluding more recent breakthroughs in LLM ability (e.g., GPT-4.5, DeepSeek-V3). (4) Although the qualitative sample under Stage Two (*n* = 8) was purposively selected by the author from their academic network, this stage was used for depth of analysis rather than for generalizability. The study validated its findings through thick description and strong consistency with Stage One, and achieved theoretical saturation, as no new conceptual meaning emerged beyond the sixth participant. However, the perspectives of younger users, including undergraduate students, should be explored further, as their patterns of reliance, perceived risk, and metacognition may differ from those of professionals. Although DeepSeek is positioned as an open-source alternative, its accessibility is shaped by governance structures, model versioning, and varying degrees of transparency across deployments. Differences in documentation quality, update stability, and institutional support may limit consistent adoption, for example, when settings require accountability, data protection, or pedagogical reliability. These differences could affect both reproducibility and trust compared with more commercially governed models.

#### 4.7.3 Future trajectories

Future research should target the following directions to advance AI in education further. (1) Implement long-term, longitudinal research that investigates AI effects on metacognition, critical thinking, and learning achievement. (2) Create AI systems possessing embedded SRL competency, enabling goal setting, monitoring, and reflection in real time. (3) Encourage teacher-AI collaboration for feedback, grading, and one-on-one learning with sustained academic rigor. (4) Extend research to multilingual and non-Western environments to guarantee equal and culturally sensitive AI adoption. (5) Accept interdisciplinary approaches combining education, cognitive science, behavioral science, data science, and policy design to create technically robust, pedagogically critical, and ethically grounded AI systems. Embraces.

## 5 Conclusion

This mixed-methods overview demonstrates that the integration of ChatGPT and DeepSeek at the tertiary level is more than a technological advance—it is a paradigm-busting change in cognitive, ethical, and pedagogical frameworks for contemporary learning. By combining 51 studies and eight qualitative findings across six thematic areas and three cross-cutting trends, the review confirms that both these generative AI models have complementary strengths: ChatGPT facilitates creativity, responsiveness, and generative fluency, while DeepSeek offers analytical rigor, domain-specific accuracy, and formative reasoning.

Despite these advantages, the adoption of GenAI poses a significant risk. Hallucination-based inaccuracies, moral ambiguities, and metacognitive stasis were perennial challenges, especially in unstructured educational settings. These challenges underscore the need to create AI-augmented learning ecosystems centered on human-AI collaboration, ethical education, and metacognitive scaffolding rather than on full automation.

The findings necessitate a reimagined learning environment where GenAI is embedded in adaptive, evidence-based, discipline-specific, learner-profile-aligned, and culturally situated approaches. Integration in this manner enables institutions to strike a balance between innovation and integrity, personalization and fairness, and scalability and human insight.

Ultimately, the future of GenAI in higher education necessitates co-designed, multidisciplinary, and world-informed practices and research. ChatGPT and DeepSeek are not competitive technologies but synergistic cognitive co-agents that can enhance creativity, precision, ethical reasoning, and reflective learning. With responsible application, they will catalyze a wiser, more inclusive, and trans figurative higher education culture.

## Data Availability

The raw data supporting the conclusions of this article will be made available by the authors, without undue reservation.
